# Blood Pressure and Heart Rate Response to Orthostasis in Somali Americans

**DOI:** 10.21203/rs.3.rs-4925722/v1

**Published:** 2024-10-21

**Authors:** Ian Greenlund, Joshua Bock, Nivash Govindan, Dimitrios Kantas, Prachi Singh, Naima Covassin, Virend Somers

**Affiliations:** Mayo Clinic Rochester: Mayo Clinic Minnesota; Mayo Clinic Minnesota; Cook County Health; Mayo Clinic Minnesota; Pennington Biomedical Research Center; Mayo Clinic Minnesota; Mayo Clinic Department of Cardiovascular Medicine

**Keywords:** Health Disparities, Autonomic, Reactivity, Cardiovascular Risk

## Abstract

**Purpose:**

Cardiovascular health disparities are present in African Americans, but it remains unknown whether this phenomenon affect Somali Americans. Study of Somali Americans is warranted due to distinct genetic and cultural differences from African Americans of western African ancestry. Orthostatic hemodynamic responses have implications for cardiovascular risk, especially among African American females. We sought to examine race and sex differences in systolic (SAP) and diastolic (DAP) arterial pressure and heart rate (HR) responsiveness to standing. We hypothesized that SAP, DAP, and HR change from supine to standing position would be higher in Somali Americans.

**Methods:**

We studied blood pressure and HR responsiveness in 139 (70 Somali; age: 29±10 years, 69 White; age: 31±9 years) participants. Supine SAP, DAP, and HR were measured after at least five minutes of supine rest, and again after one minute of standing. SAP, DAP, and HR change was compared between groups.

**Results:**

ΔSAP and ΔDAP were similar between groups (race × sex: p>0.05). However, HR responsiveness to orthostasis varied between race and sex comparisons (race×sex: p=0.011). Somali females exhibited an augmented HR response to orthostasis compared to White females (Δ19±13 vs. 11±9 beats/min, p=0.005) and Somali males (Δ19±13 vs. 12±9 beats/min, p=0.020).

**Conclusion:**

ΔHR to standing is augmented in young female Somali Americans. These findings highlight an early potential impairment in hemodynamic regulation that may heighten future cardiovascular risk. Further work is warranted to identify the potential autonomic nervous system underpinnings that may contribute to potentiated orthostatic responses and cardiovascular risk in Somali American females.

**Clinical Trial Registration:**

www.clinicaltrials.gov; unique identifier, NCT04124848; NCT05411029; NCT03308578.

## INTRODUCTION

Many American minority populations exhibit significant health inequalities compared to the White majority population, including African Americans [1], South Asians [2], and Native Americans [3]. Somali Americans are a growing minority population in the United States, especially within Minnesota, Ohio, and Washington [4]. Given that the bulk of Somali immigration to the United States only occurred in the mid to late 1990s [4], little is known of the presence and the risk of health disparities in this community. Studies of the Somali diaspora to other countries such as Norway have shown Somali immigrants often present with elevated body mass index (BMI) [5] and cardiometabolic risk [6] compared to the native Norwegian population. Cardiometabolic dysfunction is comorbid with cardiovascular disease (CVD) risk, and it is well-established that CVD is more common in African Americans [7], who appear to be at greater risk for CVD including hypertension [8], coronary artery disease [9], and heart failure [10]. However, it is unclear whether these CV health disparities are also relevant to the Somali American community. This is due to distinct genetic differences between African Americans (who are mainly of West African origin) and Somali Americans (East African descent) [11]. Therefore, there is a need to identify potential CVD mechanisms within this growing American minority population to aid with disease management and treatment.

Established CVD mechanisms include autonomic dysfunction, heightened sympathetic drive, and impaired heart rate variability [12, 13]. African Americans who are overweight manifest with heightened sympathetic drive, especially among females [14]. Further, it is suggested that normotensive African Americans also tend to display higher vasoconstrictor activity compared to White adults [15]. Collectively, heightened sympathetic outflow appears as an influential for increased hypertension (HTN) risk. Orthostatic stress is a robust activator of the autonomic nervous system [16], with ecological utility given are humans subject to orthostatic stress daily, and are upright for a significant portion of day. Importantly, heightened activation or reactivity to orthostasis may also contribute to increased HTN risk later in life [17]. However, whether male and female Somali Americans differ in systolic (SAP) and diastolic (DAP) arterial pressure and heart rate (HR) responsiveness to orthostasis compared to White adults remains to be determined.

The primary purpose of the present study was to compare systolic (SAP) and diastolic (DAP) arterial pressure and HR responses to orthostasis between Somali Americans and White adults. The secondary aim was to examine within and between race and sex differences in cardiovascular responsiveness to orthostasis. We hypothesized that Somali Americans would exhibit higher SAP, DAP, and HR changes following orthostasis compared to White adults.

## METHODS

### Participants

Study participants were analyzed from both completed and ongoing studies at Mayo Clinic–Rochester. The Mayo Clinic Institutional Review Board approved all testing procedures, which conformed to the guidelines contained with the Declaration of Helsinki. Investigators detailed all procedures to participants, allowed for questions and discussion, and obtained written and consent from all participants. Diagnosed disease and current medications were documented by self-report upon study enrollment if applicable.

Participants were requested to abstain from alcohol and caffeine consumption, and regular exercise for a minimum of 24 hours prior to laboratory arrival. Exclusion criteria for female participants included pregnancy. Adults (≥ 18 years old) who self-identified as Somali, Somali-American, or Somalian, or White, Caucasian, or European-American were recruited from the patient population at Mayo Clinic, Rochester, Minnesota, and the surrounding communities. A total of 70 Somali (34 females) and 69 White (32 females) participants were included in the present study.

### Study Design

Research study participants reported to the Mayo Clinic–Clinical Research and Trials Unit for a brief out-patient study visit. The trained nursing staff completed study intake and collected body anthropometrics (e.g., height and weight). A study team member completed a comprehensive health history interview with each patient to document any cardiovascular, pulmonary, endocrine, metabolic, or renal diagnosed disease/disorder, and relevant prescribed medications. The study team member then completed the orthostatic blood pressure (BP) and HR test.

### Measurements

#### Clinical and Orthostatic Blood Pressure and Heart Rate

Three seated clinical BP measurements were obtained following a 10-minute period of quiet rest. BP and HR were obtained in the supine and standing position with a Phillips BP monitor (MX450, Amsterdam, Netherlands). A measurement was taken after at least five minutes of quiet, supine rest, and again after one minute of quiet standing. The brachial BP cuff was placed on the patient’s non-dominant arm.

#### Analytic Plan and Statistical Analysis

Changes in BP and HR between supine and standing positions were compared between Somali and White study participants. All data were analyzed statistically using commercial software (SPSS 28.0; IBM SPSS, Armonk, NY). Assumption of normality tests were conducted on each variable of interest. Using z-score normalization of variable skewness, all primary outcome variables of BP and HR were within 3 standard deviations of the mean, indicative of normal data distribution. We utilized a 2×2 univariate analysis of variance to determine race (i.e., Somali vs. White) and sex (i.e., male vs. female) differences in BP and HR response to orthostasis. The Chi-Square Test of Independence was used to compare disease prevalence and medication usage between race and sex groups. Data are presented as mean ± standard deviation and number (percentage) as appropriate. Significance level was set as α ≤ 0.05. Effect sizes for change in BP and HR (partial eta squared; η_p_^2^) are reported.

## RESULTS

Table 1 presents group characteristics stratified by race (Somali vs. White) and biological sex (male vs. female). Age, sex, and BMI were similar between Somali and White study participants (p > 0.05 for all). The majority of the study sample was free of diagnosed disease. However, diagnosed cardiovascular disease (e.g., hypertension) exhibited higher prevalence in males, regardless of race, while metabolic/endocrine disorders (e.g., diabetes, thyroid dysfunction) were higher in Somali males. Similarly, the majority of our study sample was free of regular medication use, but on average, White females were more likely to report prescription medications. Angiotensin II antagonists were prescribed more in Somali males relative to other race and se Last, birth control use was higher in White females than Somali females.

**Figure 1:**
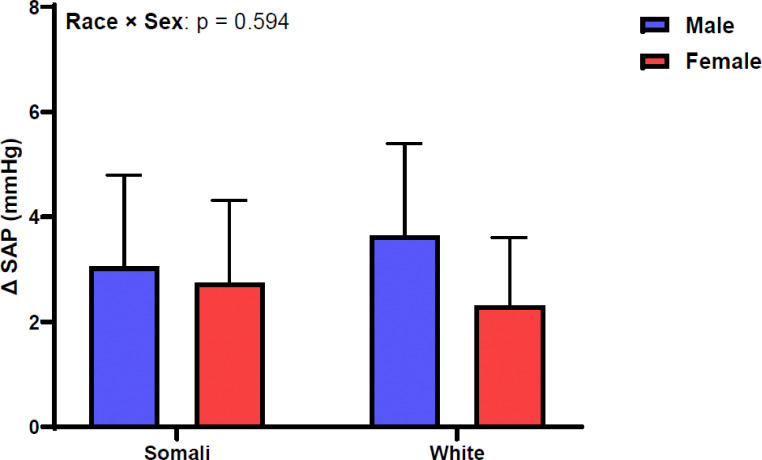


**Figure 2:**
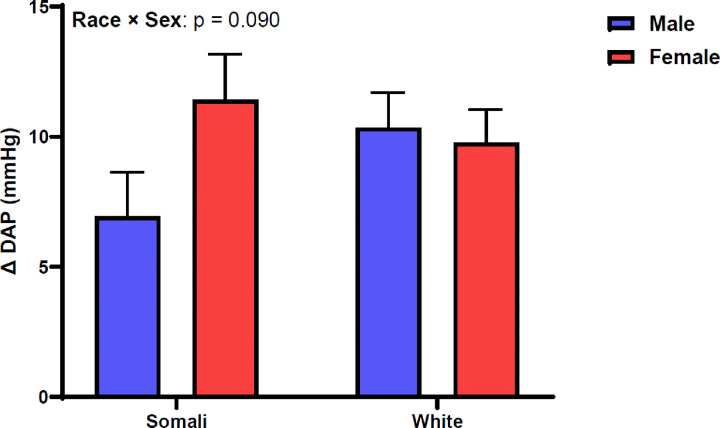


**Figure 3:**
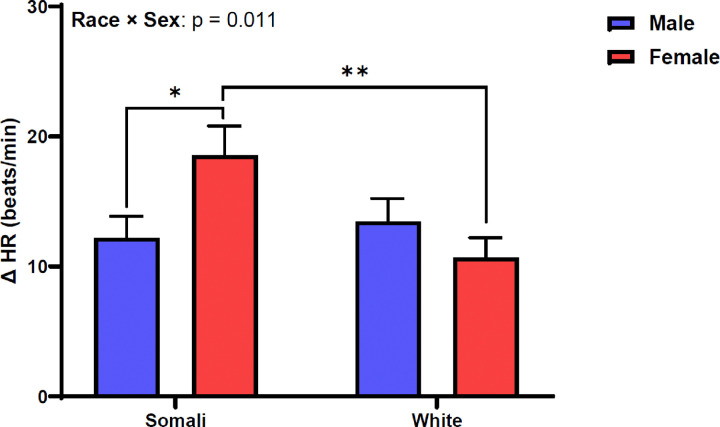


Figure 1 shows SAP change between supine and standing positions. Change in SAP was similar between male and female Somali and White participants (race × sex: p = 0.594, η_p_^2^ = 0.004). Figure 2 depicts DAP change following 1-minute orthostatic challenge. Similar to SAP, DAP did not differ by race or sex in our study sample (race × sex: p = 0.090, η_p_^2^ = 0.021). Figure 3 demonstrates HR responsiveness to 1-minute of orthostatic stress. Change in HR following standing differed between male and female Somali and White study participants (race × sex: p = 0.011, η_p_^2^ = 0.047). Post hoc analysis revealed that HR reactivity to standing was higher in Somali females compared to Somali males (19 ± 13 vs. 12 ± 9 beats/min, p = 0.020). Further, the HR increase to standing was also higher in Somali females compared to White females (19 ± 13 vs. 11 ± 9 beats/min, p = 0.005).

## DISCUSSION

The present study investigated BP and HR responsiveness to standing in male and female Somali and White study participants. We report two novel findings. First, SAP and DAP responsiveness to standing was not different between racial or sex comparisons. Second, and despite the similar BP responses, HR responsiveness to standing differed between groups. Specifically, Somali females exhibited augmented HR responsiveness to standing compared to both Somali males and White females. This phenomenon was present in a large sample of Somali and White adults of similar age, BMI, and sex distribution. These findings suggest differences in autonomic cardiac control in Somali females, which may place this group at increased CVD risk.

While BP may modestly change with an orthostatic challenge via changes in blood distribution and peripheral sympathetic outflow, HR changes (often increased) represent an effort to maintain cardiac output and BP. Standing [18], head-up tilt [19], or lower body negative pressure (LBNP) [20] elicit reproducible increases in HR and decreased heart rate variability indicative of reduced cardiac vagal activation. However, augmented HR reactivity to orthostasis is related to future CVD risk [21]. Indeed, in our sample of Somali Americans, HR reactivity to one minute of quiet standing was highest in Somali females compared to both White females and Somali males. These data are comparable to those of Hinds and Stachenfeld who saw augmented HR increases in response to LBNP in African American females compared to White females, together with higher plasma norepinephrine at presyncope [17]. While such mechanisms may be advantageous during early life by offsetting issues such as orthostatic intolerance (common in White females), they may also be indicative of autonomic dysfunction/hyperactivity. This phenomenon, greater sympathetic drive, may conceivably place Somali females at increased risk for HTN and future end-stage CVD compared to White females.

Orthostatic BP assessment exhibits broad utility in regard to impaired BP regulation [22]. More commonly, orthostatic BP is used to examine hypotension in individuals who fail to mitigate a fall in BP upon standing [23]. Conversely, BP may also increase during an orthostatic challenge. In a large sample of over 1,200 adults from the Hypertension and Ambulatory Recording Venetia Study, participants who increased BP during orthostatic challenge by > 6.5 mmHg (SAP) exhibited a nearly two-fold increased risk of major cardiovascular events compared to normal BP responders to standing [24]. While ΔSAP was similar across groups, DAP reactivity approached significance (Interaction: p = 0.090) perhaps driven by attenuated DAP increase in Somali males. While caution is warranted so as to not overinterpret these data, arterial stiffness may also be influential in this relationship in both sexes. Reduced basal vascular compliance may facilitate greater orthostatic responses from poor elastic recoil to preserve diastolic flow and result in an increased standing tachycardic response. In previous data from our laboratory, Somali Americans exhibited heightened arterial stiffness via 24-hour ambulatory BP monitoring compared to White adults (*data pending publication*). Further work is needed to elucidate potential sex differences in arterial stiffness within the Somali American community.

Measuring orthostatic BP and HR for clinical practice can be utilized to rule out potential masked HTN, which is defined as an elevated mean daytime ambulatory BP in the presence of normal or non-elevated clinic blood pressures [25]. Epidemiological evidence from the Masked HTN study shows that 15% of the majority White study sample exhibited masked HTN [26]. In comparison, in a sample of 972 African Americans from the Jackson Heart Study, masked HTN prevalence was over two-fold higher at 34% of the study sample [27, 28]. Such a phenomenon may help explain cardiovascular health disparities within the African American community and may extend to Somali Americans. While we cannot comment on masked HTN in our younger Somali American population, the observed evidence within the African American community, in conjunction with our orthostatic HR responsiveness in Somali females, suggests that masked HTN may be prevalent within the Somali American community. Further, examination of orthostatic BP and HR in the Somali American community may offer utility to alleviate CVD burden within these populations via earlier intervention (behavioral or pharmacological).

While this hypothesis-generating study has multiple strengths including large sample size and majority disease free participants, it is not without limitations. Presumed autonomic dysfunction in Somali females should be confirmed using gold-standard techniques like microneurography in a Somali American sample free of disease and medication use. Indeed, a percentage of our sample exhibited diagnosed disease, but this limitation is mitigated by inclusion of a majority of disease- and medication-free Somali and White study participants.

Growing evidence suggests Somali Americans may be at increased risk for CVD, but investigation of potential CVD mechanisms in this population is completely absent. Our present study shows heightened HR reactivity to a one-minute orthostatic challenge (standing) in younger Somali females. These findings suggest that while younger Somali females may be at lower risk for orthostatic intolerance from hypothesized higher sympathetic drive, this may contribute to greater CVD risk with age.

## Figures and Tables

**Table 1:** Participant Demographics

	Somali (n = 70)		White (n = 69)		P-Value		
	Male	Female	Male	Female	Race	Sex	R×S
N	36	34	37	32	---	0.796	---
Age (yrs)	30± 10	27 ± 9	33 ± 9	29 ± 8	0.135	0.019	0.799
BMI (kg/m^2^)	29 ± 7	27 ± 8	29 ± 4	26 ± 6	0.580	0.035	0.743
Seated SAP (mmHg)	122 ± 13	112 ± 10	124 ± 12	113 ± 11	0.353	< 0.001	0.665
Seated DAP (mmHg)	75 ± 9	74 ± 7	77 ± 12	73 ± 9	0.710	0.128	0.257
Seated HR (beat/min)	68 ± 13	71 ± 12	68 ± 11	70 ± 11	0.679	0.206	0.841
Disease, n (%)							
None	22 (61)	30 (88)	32 (86)	25 (78)	0.963	0.091	0.199
Cardiovascular	3 (8)	0 (0)	2 (5)	0 (0)	0.661	0.030	0.162
Pulmonary	6 (17)	3 (9)	7 (19)	3 (9)	0.779	0.135	0.510
Metabolic/	8 (22)	1 (3)	2 (5)	4 (13)	0.429	0.245	0.041
Endocrine							
Renal	1 (3)	0 (0)	0 (0)	0 (0)	0.319	0.340	0.410
Medication, n (%)							
None	29 (81)	32 (94)	36 (97)	21 (66)	0.455	0.151	< 0.001
AngII Antagonist	3 (8)	0 (0)	0 (0)	0 (0)	0.082	0.096	0.032
Biguanide	5 (14)	1 (3)	0 (0)	3 (9)	0.312	0.850	0.073
β- Antagonist	1 (3)	0 (0)	1 (3)	0 (0)	0.568	0.620	0.820
Glucocorticoid	1 (3)	0 (0)	0 (0)	0 (0)	0.319	0.340	0.410
Hormone Replacement	2 (6)	0 (0)	0 (0)	0 (0)	0.082	0.620	0.309
Oral Contraceptive	---	1 (3)	---	9(28)	0.023	---	---
Statin	2 (6)	1 (3)	0 (0)	1 (3)	0.317	0.918	0.567

Results are means ± SD or reported prevalence (percentage); SAP, Systolic Arterial Pressure; DAP, Diastolic Arterial Pressure; HR, Heart Rate, AngII, Angiotensin II; BMI, body mass index.
